# Chemical Composition and* In Vitro* Antioxidant, Cytotoxic, Antimicrobial, and Larvicidal Activities of the Essential Oil of* Mentha piperita* L. (Lamiaceae)

**DOI:** 10.1155/2017/4927214

**Published:** 2017-01-01

**Authors:** Ryan da Silva Ramos, Alex Bruno Lobato Rodrigues, Ana Luzia Ferreira Farias, Ranggel Carvalho Simões, Mayara Tânia Pinheiro, Ricardo Marcelo dos Anjos Ferreira, Ledayane Mayana Costa Barbosa, Raimundo Nonato Picanço Souto, João Batista Fernandes, Lourivaldo da Silva Santos, Sheylla Susan Moreira da Silva de Almeida

**Affiliations:** ^1^Laboratory of Pharmacognosy and Phytochemistry, Federal University of Amapá, Highway Juscelino Kubitschek, Km 02, Macapá, AP, Brazil; ^2^Laboratory of Arthropoda, Federal University of Amapá, Rodovia Juscelino Kubitschek, Km 02, Jardim Marco Zero, Macapá, AP, Brazil; ^3^Department of Chemistry, Federal University of São Carlos, Washington Luis Highway, Km 235, SP-310, 13565-905 São Carlos, SP, Brazil; ^4^Laboratory of Chemistry, Federal University of Pará, Rua Augusto Corrêa, 01-Setor Básico, 66075-110 Belém, PA, Brazil

## Abstract

The essential oil was obtained by hydrodistillation and the identification and quantification of components were achieved with the use of GC-MS analysis. The antioxidant activity was evaluated by the method of sequestration of DPPH. Essential oils were used for study the cytotoxic front larvae of* Artemia salina*. In the evaluation of the antimicrobial activity of essential oils, we employed the disk-diffusion method. The potential larvicide in mosquito larvae of the third stage of development of* Aedes aegypti* to different concentrations of essential oils was evaluated. The major compounds found in the essential oils of* M. piperita* were linalool (51.8%) and epoxyocimene (19.3%). The percentage of antioxidant activity was 79.9 ± 1.6%. The essential oil showed LC_50_ = 414.6 *μ*g/mL front of* A. saline* and is considered highly toxic. It shows sensitivity and halos significant inhibition against* E. coli*. The essential possessed partial larvicidal efficiency against* A. aegypti*.

## 1. Introduction

The International Standard Organization (ISO) defines the essential oils (EO) as volatile products extracted from plants by steam distillation, in most cases. They are usually complex mixtures of volatile, lipophilic, liquid, and odoriferous substances and associated with various functions necessary for the survival of the plant in its ecosystem, playing a key role in the defense against microorganisms and predators and in attracting insects and other fertilizing agents. In traditional medicine, essential oils have a long tradition of use [1, 2].

The Lamiaceae family has potential in obtaining EO and it plays and important role in various sectors of the economy, for example, the pharmaceutical industry, perfumery, cosmetics, and food. Several biological functions are associated with species by popular medicine, used for the treatment of burns, headache, colic, fever, reports of antiflu activity, antiemetic, carminative, insecticide, repellent, antibacterial, and combat intestinal parasites [3–5].

The genus* Mentha*, popularly known in Brazil as “hortelãs, hortelã-pimenta, menta, menta-inglesa, hortelã-apimentada, hortelã das cozinhas and sândalo,” includes 25–30 species that are known mainly due to the characteristic and refreshing taste. It is native to Europe and was brought by settlers to Brazil, where it is cultivated as medicinal plant in gardens [6, 7].

The literature logs its spasmolytic activities: antivomitive, carminative, stomachic and anthelmintic, topical antibacterial, antifungal, and antipruritic. In the preliminary phytochemical analysis of the essential oil, there was the presence of the main constituents: the majority were menthol, menthone, and menthofuran compounds which were responsible for pleasant odor [8–11].

The Amazon region with its immense biodiversity offers a great potential for the discovery of new flavors and products. However, there is great scarcity of chemical and biological activities information associated with plant species due to deficient support in research work in biotechnology. The aim of this study is to evaluate the chemical composition and* in vitro* antioxidant, cytotoxic, antimicrobial, and larvicidal activities of the essential oil of* Mentha piperita* L. (Lamiaceae).

## 2. Material and Methods

### 2.1. Collection of Plant Material

The species were collected in the city of Macapá, AP, under the coordinates 00°02′23′′S and 51°06′29′′W and, later, sent to the Herbarium of the Institute of Scientific and Technological Research of the State Amapá, IEPA, for taxonomic identification procedures and preparation of voucher specimen, under the registration number RRS 001.

### 2.2. Obtaining Essential Oil

The extraction of essential oils was held in Pharmacognosy and Phytochemistry Laboratory of the Federal University of Amapá (UNIFAP). The leaves were treated (washed and dried) for extracting their essential oils. The essential oil was obtained by hydrodistillation (temperature 100°C) in a Clevenger-type apparatus for 4 h [12].

### 2.3. Chromatographic Analysis (GC-MS)

The essential oil analysis was performed by gas chromatography coupled to mass spectrometry (GC-MS) at the Federal University of São Carlos (UFSCar). We used Shimadzu equipment, GCMS Shimadzu QP 5000, and employed a capillary column of fused silica OPTIMA® 5–0.25 *μ*m, 30 m in length, and 0.25 mm internal diameter and nitrogen as a carrier gas. The gas chromatograph operating conditions were as follows: internal column pressure of 67.5 kPa, split ratio 1 : 20, the gas flow in column 1.2 mL/min. (210°C), injector temperature 260°C, and the temperature detector or the interface (GC-MS) of 280°C. The initial column temperature was 50°C followed by an increase of 6°C/min. up to 260°C and maintained constant for 30 min. The mass spectrometer was programmed to perform readings in a range 29–400 Da, 0.5 s intervals with ionization energy of 70 eV.

### 2.4. Component Identification

The identification of the constituents of essential oils was performed in comparison with the Kovats Index (KI) of the homologous series of n-alkanes (C_8_–C_26_ + C_28_) and literature [13]. Further identification was made by combining their mass spectra with the registered and stored equipment in the Wiley version 2.5/MBP GC-MS system library.

### 2.5. Analysis of Antioxidant Activity

Evaluation of antioxidant activity was held in Pharmacognosy and Phytochemistry Laboratory of the Federal University of Amapá (UNIFAP). Based on the methodology proposed, we used 2,2-diphenyl-1-picrylhydrazyl (DPPH) with some modifications [14–16].

A methanol solution of DPPH at the concentration of 40 *μ*g·mL^−1^ was prepared. The essential oils were diluted in methanol concentrations (5, 1, 0.75, 0.50, and 0.25 mg·mL^−1^). For the evaluation, they were added into a test tube of 2.7 mL of the stock solution of DPPH, followed by addition of 0.3 mL of the essential oil solution. Meanwhile, the negative control was prepared, this being a mixture of 2.7 mL of methanol and the methanolic solution of evaluated compounds. After 30 minutes, the readings were taken in a spectrophotometer (Biospectro SP-22) at a wavelength of 517 nm. For comparison, ascorbic acid was evaluated.

### 2.6. Toxicity Test

The toxicity test against* A. salina* Leach was held in Pharmacognosy and Phytochemistry Laboratory of the Federal University of Amapá (UNIFAP). Initially, 250 mL of synthetic sea salt solution (35.5 g/L) was prepared for incubation of 25 mg of* A. salina* eggs, which were exposed to artificial light in 24-hour period for onset of lava (metanauplius); then, the metanauplii were separated and placed in the dark for 24 h period to reach nauplii stage [17–19].

The solution was prepared to contain 62.5 mg of essential oil, 28 mL of synthetic sea salt solution, and 2 mL of dimethylsulfoxide (DMSO) to facilitate its solubilization. Subsequently, at the end of the period in the dark, they were selected and divided into 7 groups of 10 subjects in each test tube. Each group has added a solution of the rate (3125, 2500, 1250, 625, 250, 25, and 2.5 *μ*L). The volume of 5 mL with synthetic sea salt solution was reached, to yield final solutions with the following concentrations of 1250, 1000, 500, 250, 100, 10, and 1 *μ*g/mL. Thereby, the groups were designated according to their respective concentration and all tests were performed in triplicate. At the end, the number of nonsurvivors were counted, to determine LC_50_ through probit analysis of the SPSS® software.

### 2.7. Antimicrobial Activity

 The microbiological activity of essential oils was studied in the Microbiology Laboratory of the Faculty Estacio SEAMA. It used two strains of bacteria, one Gram-positive (*Staphylococcus aureus*, ATCC 25923) and another,Gram-negative (*Escherichia coli*, ATCC 25922), being performed according to the rules and procedures of Clinical and Laboratory Standards Institute (CLSI) [20]. The test for evaluation of the antibacterial activity was performed in triplicate.

#### 2.7.1. Culture Media

The medium Mueller-Hinton (MH) agar was used for antimicrobial activity assays and prepared according to manufacturer's instructions, followed by release of 25 mL of agar medium per Petri dish of 90 × 15 mm.

#### 2.7.2. Bacterial Suspension and Inoculum

 The growth of Mueller-Hinton agar, after incubation for 24 hours at 37°C, peaked up from 2 to 4 colonies in 1 mL of sterile 0.85% saline solution, until a turbidity similar to the 0.5 McFarland scale obtained a final concentration of 1,5 × 10^8^ UFC/mL.

#### 2.7.3. Evaluation of Antimicrobial Activity

To evaluate the antimicrobial activity of essential oils, we used the disk agar diffusion method. Each bacterial suspension was plated (in triplicate) with the aid of a stereodisposable swab across the surface of Mueller-Hinton agar. A solution was prepared with a solubilized concentration of 250 mg/mL with Tween 80 and then, dilutions were held to obtain concentrations of 100, 50, and 10 mg/mL. After that, it was soaked on filter paper discs (Whatman, type 3) of 6 mm diameter each 10 L concentrations, respectively. After incubation of the plates at 35°C for 24 hours, a reading of the results by measuring the halo formed around disks containing the oil was performed. The result of each dilution, the average of the three measurements, and susceptible halo of an equal size or greater than 8 mm diameter were considered [21, 22].

The disk-diffusion experiment was controlled by using disks containing the antibiotics reference: cefoxitin, oxacillin, and gentamicin to verify the sensitivity of the test microorganism and negative controls were impregnated with discs containing water and Tween 80.

### 2.8. Larvicidal Activity

#### 2.8.1. Larvae

The larvae of* A. aegypti* used in the bioassays were from the colony maintained at the Insectarium of the Arthropoda Laboratory at the Federal University of Amapá, all of F_6_ generation, in the third young stadium, Macapá, Amapá lineage.

#### 2.8.2. Bioassays

The biological tests were conducted in a room (3 m × 4 m) with controlled climatic conditions: temperature 25 ± 2°C, relative humidity of 75 ± 5%, and photoperiod of 12 hours, located at Arthropoda Laboratory of the Federal University of Amapá, Macapá.

The methodology followed the standard WHO protocol with modifications to the test container [23, 24]. After analyzing preliminary test series, the concentrations were selected: 500, 400, 300, 200, and 130 ppm.

A stock solution was prepared with essential oil, presolubilized in Tween 80, and dissolved in 93 mL of water to obtain a concentration of 1500 ppm. From this solution, a dilution series was prepared to obtain the concentrations of solutions 500, 400, 300, 200, and 130 ppm. For each replicate of a treatment, 10 larvae were used, pipetted into a beaker containing 100 mL of distilled water. Then, the larvae were removed from the beaker into the test container, thus minimizing the time between the preparation of the first and last sample. The safety of the solvent was observed in the concentration used, with the same present also in control of the replicas. During the experiment, the average water temperature was 25°C. After 24 and 48 hours, dead larvae were counted, being considered as all those unable to reach the surface. The data obtained from the mortality (%) × concentration (ppm) were analyzed by SPSS in probit graph to determine the lethal concentration that causes 50% mortality of the population (LC_50_).

### 2.9. Statistical Analysis

Statistical analysis was performed by analysis of variance (ANOVA). The significant differences between averages were determined by Tukey' test.

## 3. Results and Discussion

The yield of essential oil obtained from the hydrodistillation process of the species* M. piperita* was 0.08% (m/m). In total, 15 components were identified, representing 94.9% of the total amount. Compounds identified in the essential oil of the species were as follows: thuja-2,4(10)-diene (0.3%), verbenene (2.6%), *β*-pinene (3.8%), mentha-2,8-diene (0.4%), *β*-ocimene (0.4%), linalool (51.8%), epizonarene (0.6%), epoxyocimene (19.3%), sesquiphellandrene (9.4%), cadinene (4.0%), and germacrene B (2.3%).

To the best of our knowledge, this is the first record of the chemical composition of the* M. piperita* essential oil collected in the city of Macapá, Brazil. The chemical composition of the essential oil (Table 1) shows a high percentage of oxygenated monoterpenes (71.1%).

**Figure 1:**
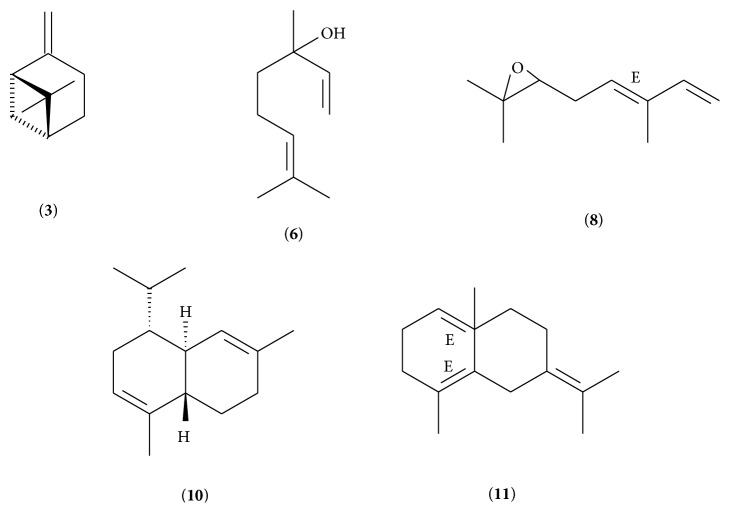
Chemical structure of major compounds identified in the essential oil.

The essential oil of* M. piperita* contains compounds with properties of biological interest. Some authors believe that the linalool has antifungal, antimicrobial [25, 26], antitumor [27], antimutagenic [28], analgesic, antispasmodic [29], anti-inflammatory [30], antiparasitic [31], antiplatelet, and antioxidant activity [32]. The chemical structure of major compounds identified in the essential oil of the species is shown in Figure 1.

Other studies of Sartoratto et al. [33] showed that the essential oils obtained from the dried leaves of the species* M. piperita* have the major compound linalool (51.0%) in its chemical constitution. The results are in agreement with previous studies of* M. piperita* essential oil collected in Brazil, characterizing linalool (51.8%) as the most important major component. However, several studies of the chemical composition found that, in other countries, menthol is the major component. The diversity of chemical compounds in the essential oil of the species is attributed to soil factors, biosynthetic, and collection time [34–38].

### 3.1. Antioxidant Activity

The antioxidant activity (ability or antioxidant potential) is a widely used parameter to characterize different biological materials. This activity is related to compounds capable of protecting a biological system against the harmful effects of processes or reactions that cause excessive oxidation involving reactive oxygen species [39, 40].

The vast majority of compounds with antioxidant properties have a molecular structure having at least one aromatic ring and a hydroxyl group, including phenols, flavonoids and isoflavones, esters, lignin, coumarins, flavones, and oligomeric proantocianidinas. In mixtures of these compounds, an antioxidant arrangement is produced that may act by different mechanisms to give an effective defense system against free radicals. Alcohols are the second class of oxygenated monoterpenes, which comprise the essential oil, and more active in oxidizing activities [41].

The mean values of percentage of antioxidant activity (%AA) of essential oils are shown in Table 2.

The species* M. piperita* has significant antioxidant activity and *p* < 0.0001 in the concentration of 100 mg·mL^−1^ with % AA 79.9 ± 1.6. The antioxidant activity is associated with the presence of linalool major compound (51.80%). The inhibitory concentration (IC_50_) by linear regression presented a value of 0.54 mg·mL^−1^, showing the strong coefficient correlation (*R* ^2^) of 0.9770. On the other hand, if polar compounds, such as ascorbic acid (IC_50_ 0.12 mg·mL^−1^) compared to the essential oil, were only tested by it, they would be considered weak antioxidants.

The results of antioxidant activity are similar to previous studies of Derwich et al. [35] % AA values ≥ 81.09 in the concentration of 150 *μ*g·mL^−1^ and IC_50_ ≥ 53.67 *μ*g·mL^−1^. However, previous studies of essential oil species showed menthol as the major compound, in which the radical scavenging activity is associated with the presence of compounds menthone and menthol, both compounds with the presence of the hydroxyl radical (-OH). The species* M. piperita* is described by several authors as a potential antioxidant, and there are also reports of cytotoxic activity. Some authors describe the possible elimination of monoterpene ketones (menthone and isomenthone) and 1,8-cineole in changing the chemical composition of the species [35].

### 3.2. Toxicity Test

Table 3 refers to the average mortality readings performed within 48 h, in the cytotoxic activity of the essential oil of* M. piperita*. The results are significant and expressed in percentage of mortality (%).

The essential oil of* M. piperita* showed LC_50_ 414.6 *μ*g·mL^−1^ and correlation coefficient of 0.7443, confirming its moderate toxicity front of* A. salina*. The oil of* Mentha *spp. has a calming pain action and is clinically used as an ingredient in many analgesics creams and in the treatment of arthritis and other musculoskeletal conditions. The chemical compound menthol relieves discomfort by afferent modulator, causing pain impulses, as part of an astringent effect, and exciting the nerves that recognize the sensation of cold.

Essential oils were characterized as good bioactive agents, because of oils and extracts from plants presenting LC_50_ values below 1000 *μ*g·mL^−1^ and previous studies by Andrade et al. [41], Meyer et al. [42], and Nascimento et al. [43] considered them as bioactive. Specifically, oils and plant extracts have degrees of toxicity against larvae of* A. salina. *According to the intervals, they demonstrated activity at or below 100 *μ*g·mL^−1^ and were considered as having a strong cytotoxic activity. LC_50_ values between 100 and 500 *μ*g·mL^−1^ were considered to be moderately toxic. LC_50_ values between 500 and 1000 *μ*g·mL^−1^ were considered as weak cytotoxic activity, while LC_50_ values greater than 1000 *μ*g·mL^−1^ were categorized as nontoxic [44].

Several dangerous and even fatal side effects have been reported about herbal products. These side effects may occur by several different mechanisms, including direct toxicity, contamination, and interactions with drugs and other herbs. The essential oil of* M. piperita* is associated with side effects such as heartburn, nausea, vomiting, allergic reactions, flushing, and headaches.

### 3.3. Antimicrobial Activity

Natural products have become an excellent therapeutic alternative, where the research for plant compounds that have some pharmacological property has intensified significantly in recent years. That has awakened new looks to the Amazon region, because its flora is estimated at more than 55,000 species and less than 1% of it has been scientifically investigated [45, 46].

A Gram-positive bacterium protects its cytoplasmic membrane with a thick cell wall. The layers of peptidoglycan prevent the passage of hydrophilic compounds due to the presence of sugars and amino acids. The outer lipoprotein membrane presents characteristics and the bacteria need to have a mechanism enabling the entry of hydrophilic compounds like sugars, amino acids, and ions. Therefore, the outer membrane has special channels called porins, which allow the passive diffusion of hydrophilic compounds [47].

The oils mechanism of action about antimicrobial effect is acting directly on the bacterial cell wall structure, denaturing and coagulating proteins. Specifically, they act by altering the permeability of the cytoplasmic membrane by hydrogen ions (H^+^) and potassium (K^+^). The amendment of the ion concentration gradients leads to deterioration of the essential processes of cell, such as transport of electrons, translocation proteins, stages of oxidative phosphorylation, and other reactions dependent enzymes, resulting in loss of chemiosmotic control of the affected cell, leading to cell death [37].

Tables 4 and 5 refer to the halo measurement results of inhibition of essential oil* M. piperita *(expressed in mm) against strains of* Staphylococcus aureus *and* Escherichia coli.*

The essential oil of* M. piperita* has submitted inhibition zone in 100 mg·mL^−1^ concentrations at 7.6 ± 0,57 mm and 10 mg·mL^−1^ with 9 ± 1.0 mm for* S. aureus* and* E. coli*, respectively. Compared with positive control and concentrations tested, there was significant difference between the inhibition zones, in which the halo in the positive control showed halo of 15 mm. The nonoccurrence of menthol may have influence on the occurrence of only one strain of bacteria, whereas linalool is also described such as potential agent in the control of bacteria.

The extract of* M. piperita* being either an essential oil or an alcoholic extract has antimicrobial properties [48]. The linalool is a major component and it contributes to the antimicrobial potential of this plant. This potential essential oil of* M. piperita* has proven in this study that the results show antimicrobial activity only for the bacteria* E. coli*.

### 3.4. Larvicidal Activity

Several studies demonstrate the activity of essential oils and plant extracts against different species of mosquitoes including* A. aegypti *[49–51]. The study of potential larvicide, insecticide, and repellent of essential oils is an alternative technology to synthetic products destined to vector control, since, to date, there is no vaccine ready for use against the four serotypes of dengue. Active agents at potential larvicide essential oils and plant extracts at LC_50_ values of <100 ppm are considereed.

The researches for vector control come showing the efficiency of the larvicidal effect of essential oils. Some studies detect the effects of the chemical composition of monoterpenes, as well as, some sesquiterpenes, which serve as repellents at toxicity significant to insects but negligible toxicity to mammals. Mixtures of these volatile low molecular weight compounds provide plants, such as* Mentha piperita*,* Citrus limon*,* Ocimum basilicum*, and* Salvia officinalis,* with their distinctive odor, which are commercially available, produced, and important ingredients for fragrance and flavor creation because of their specific sensory characteristics [52].

Table 6 refers to the average mortality readings performed in the period of 48 h of the larvicidal activity of essential oils from* M. piperita*. The results are significant and expressed in percentage of mortality (%).

According to the bioassay, the species* M. piperita* has a low potential larvicide at LC_50_ 367.6 ppm, *p* (value) < 0.01, and coefficient of determination (*R* ^2^) of 0.9980; these results are statistically significant. The study is similar to research by Kumar et al. [52]. Reduction of LC_50_ in periods of 24 or 48 hours (111.9 and 98.66 ppm) in the potential larvicide of the species was observed. The bioactivity is ineffective for the larvae, because they develop from pupas into adults in the 60–72 h period.

The essential oils extracted from various plants have been reported to have larvicidal and repellent properties against* A. aegypti *[53, 54]. Although some of the reports are available in relation to repellent potential of the* M. piperita* essential oil against insects, the research is of fundamental importance to respect the larvicidal effect evaluation of the mosquito in the preparation of a bioproduct that does not harm the environment.

The evaluation of larvicidal activity is in agreement with studies made by Pavela et al. [54]. That determined the value LC_50_ > 100 mg·L^−1^ against larvae of* Culex quinquefasciatus*, having as a major component the monoterpene which is L-(−)-menthol, in which the participation in the EO differs depending on the genotype of 20.5% to 57.3%, together with carvone (0.0 to 56.8%) and piperitenone oxide (0.0 to 31.8%).

## 4. Conclusions

The results found in this study show a great potential for use/application of the essential oil of* M. Piperita* species because it has a diversified class of chemical compounds at antioxidant, microbial, and cytotoxic activities. The information obtained in this research corroborates the use of species reported by traditional communities and literature.

Additionally, the use of these plants for the prevention and treatment of various human diseases seems reasonable and useful. The research contributes to enhancement and aggregation of the products of the Brazilian biodiversity, in the field of utilization of the species and insertion in the treatment of underlying disease in isolated regions where drugs in primary care health centers are sometimes not provided.

## Figures and Tables

**Table 1:** Major constituents of the essential oils of *M. piperita*.

Number	tR (min)	KI	Components	Peak Area (%)
1	8.88	960	Thuja-2,4(10)-diene	0.3
2	9.08	967	Verbenene	2.6
3	9.96	979	*β*-Pinene	3.8
4	10.30	983	Mentha-2,8-diene	0.4
5	13.20	1177	*β*-Ocimene	0.4
6	16.12	1096	Linalool	51.8
7	18.31	1501	Epizonarene	0.6
8	18.50	1142	Epoxyocimene	19.3
9	19.07	1522	Sesquiphellandrene	9.4
10	19.30	1538	Cadinene	4.0
11	19.85	1561	Germacrene B	2.3

			Monoterpene hydrocarbons	7.5
			Oxygenated monoterpenes	71.1
			Sesquiterpene hydrocarbons	16.3

*Total*				*94.9*

tR: retention time; KI: Kovats Index [13].

**Table 2:** Percentages results of antioxidant activity (% AA) of essential oil.

Species	Concentration (mg·mL^−1^)					
	5	1	0.75	0.5	0.25	IC_50_
*M. piperita*	79.9 ± 1.7^a^	54.9 ± 1.8^bf^	53.0 ± 0.3^cf^	47.2 ± 0.5^dg^	47.6 ± 1.04^eg^	0.54

Values (% AA) that followed the same characters do not show statistically significant differences for ANOVA (*p* < 0.05).

**Table 3:** Results of the mortality of *M. piperita* front toxicity test against larvae of *A. salina*.

Species	Concentration (*μ*g·mL^−1^)							
	1250	1000	500	250	100	10	1	LC_50_
*M. piperita*	95.4^a^	80.7^b^	60.3^c^	45^d^	20^e^	12^e^	0^f^	414.6

Different lowercase characters represent significant differences in mortality between the concentrations of the oils.

**Table 4:** Measurement results of the inhibition halos (mm) of the essential oil of *M. piperita* against strains of *Staphylococcus aureus*.

Species	Concentration			
	100 mg·mL^−1^	50 mg·mL^−1^	10 mg·mL^−1^	1 mg·mL^−1^
*M. piperita*	7.6 ± 0.57	*NA*	*NA*	*NA*

*NA*: did not present an inhibition halo.

**Table 5:** Measurement results of the inhibition halos (mm) of the *M. piperita* essential oil against *Escherichia coli* strains.

Species	Concentration			
	100 mg·mL^−1^	50 mg·mL^−1^	10 mg·mL^−1^	1 mg·mL^−1^
*M. piperita*	7.6 ± 0.57	7 ± 0.0	9 ± 1.0	7.3 ± 0.57

**Table 6:** Mortality percentage and lethal concentration (LC_50_) of *M. piperita* essential oil front *Aedes aegypti* larvae.

Species	Concentration (ppm)					
	500	400	300	200	130	LC_50_
*M. piperita*	86.6^a^	66.8^b^	40.8^c^	17^d^	3.4^e^	367.6

Different lowercase characters represent significant differences in mortality between the concentrations of the oils.
